# Defining a clinical prediction rule to diagnose bacterial gastroenteritis requiring empirical antibiotics in an emergency department setting: A retrospective review

**DOI:** 10.1007/s12664-022-01304-w

**Published:** 2023-02-08

**Authors:** Shanaz Matthew Sajeed, Michael P. De Dios, Ong Wei Jun Dan, Amila Clarence Punyadasa

**Affiliations:** 1 Department of Intensive Care Medicine, Ng Teng Fong General Hospital, Singapore, Singapore; 2 Department of Emergency Medicine, Ng Teng Fong General Hospital, 1 Jurong East Street 21, Singapore, 609606 Singapore; 3 Department of Respiratory Therapy, Ng Teng Fong General Hospital, 1 Jurong East Street 21, Singapore, 609606 Singapore

**Keywords:** Antibiotics, Bacterial, Clinical decision rule, Emergency department, Empirical, Gastroenteritis, Prediction rule, Scoring

## Abstract

**Background:**

Gastroenteritis (GE) is a non-specific term for various pathologic states of the gastrointestinal tract. Infectious agents usually cause acute GE. At present, there are no robust decision-making rules that predict bacterial GE and dictate when to start antibiotics for patients suffering from acute GE to the emergency department (ED). We aim to define a clinical prediction rule to aid in the diagnosis of bacterial GE, requiring empirical antibiotics in adult patients presenting to the emergency department with acute GE.

**Methods:**

A two-year retrospective case review was performed on all cases from July 2015 to June 2017 that included patients with acute GE symptoms referred to the ED, after which their stool cultures were performed. The clinical parameters analyzed included patient with comorbid conditions, physical examination findings, historical markers, point-of-care and radiographic tests and other laboratory work. We then used multi-variate logistic regression analysis on each group (bacterial culture–positive GE and bacterial culture–negative GE) to elucidate clinical criteria with the highest yield for predicting bacterial gastroenteritis (BGE).

**Results:**

A total of 756 patients with a mean age of 52 years, 52% female and 48% male, respectively, were included in the study. On the basis of the data of these patients, we suggested using a scoring system to delineate the need for empirical antibiotics in patients with suspected bacterial GE based on six clinical and laboratory variables. We termed this the BGE score. A score 0 – 2 points suggests low risk (0.9%) of bacterial GE. A score of 3 – 4 points confers an intermediate risk of 12.0% and a score of 5 – 8 points confers a high risk of 85.7%. A cut-off of  ≥ 5 points may be used to predict culture-positive BGE with a 75% sensitivity and 75% specificity. The area under the receiver operating characteristic (AUROC) for the scoring system (range 0 – 8) was 0.812 (95% CI: 0.780–0.843) *p*-value < 0.001.

**Conclusion:**

We suggest using the BGE scoring system (cut-off ≥ 5 points) to delineate the need for empirical antibiotics in patients diagnosed with gastroenteritis. While this is a pilot study, which will require further validation with a larger sample size, our proposed decision-making rule will potentially serve to improve the diagnosis of BGE and thus reduce unnecessary prescription of antibiotics, which will in turn reduce antibiotic-associated adverse events and save on costs worldwide.

Bullet points of the study highlights ***What is already known?***

- Infectious agents usually cause acute gastroenteritis (GE). Etiological agents can be viral, bacterial or protozoal. While the main goals of management in the emergency department (ED) are symptomatic treatment and hydration, some patients are started on antibiotics for presumptive bacterial gastroenteritis (BGE) despite the evidence guiding this practice being scant.

***What is new in this study?***

- There are no robust decision-making rules that predict bacterial GE, which dictate when to start antibiotics in patients presenting with acute GE to the ED. We aim to derive a clinical prediction rule based on both historical and laboratory markers to help predict BGE in adults.

***What are the future clinical and research implications of the study findings?***

- A clinical decision rule can help in guiding antiobiotic prescription and preventing overprescription of antibiotics. It can be further be refined and validated in future research.

## Introduction

Gastroenteritis (GE) is an umbrella term that encompasses various pathologic states of the gastrointestinal (GI) tract that manifests as abdominal pain, diarrhea and vomiting. However, it is most commonly used to denote an acute infection of the GI tract that manifests principally as diarrhea. While there is no universal definition of what constitutes a diarrheal episode, most definitions center upon a combination of the frequency, consistency and water content of the stools in question [1]. We prefer defining diarrhea in terms of consistency and specifically as stools that are either loose or of watery consistency, usually associated with increased frequency of stooling. Although diarrhea is often the commonest presentation of GE, it is important to note that GE may also have accompanying symptoms such as nausea and/or vomiting, fever and abdominal pain [2].

Infectious agents are the most common causes for acute GE. These agents give rise to diarrhea by numerous pathogenetic mechanisms that include adherence, mucosal invasion, enterotoxin production and/or cytotoxin production [2]. These mechanisms result in increased GI fluid secretion and/or decreased absorption, which produces an increased luminal fluid content that cannot be adequately reabsorbed, leading to dehydration, as well as the loss of electrolytes and nutrients.

Acute GE outbreaks are a substantial public-health concern throughout the world, especially in the developing world, due to its associated morbidity and mortality. Etiological agents can be subdivided into viral, bacterial or protozoal. The bacterial agents can be further classified as being either enteropathogenic, toxigenic or both. While the main goals of management in the emergency department (ED) are symptomatic treatment and hydration, some patients are started on antibiotics for the treatment of presumptive bacterial gastroenteritis (BGE), while only a minority actually receive stool cultures to definitively ascertain the causative agent of their GE. The guidelines of the American College of Gastroenterology (ACG) recommend stool cultures in the presence of severe diarrhea (defined as greater than six times in a 24-hour period), temperature ≥ 38.5 °C (taken orally), passage of bloody stools and persistent diarrhea, which was defined as greater than three days’ duration [3].

However, there are no robust decision-making rules that accurately predict BGE, hence allowing judicious prescription of antibiotics in patients diagnosed with acute GE to the ED, although a few have been proposed, for example, by Cadwgan et al. [4].

We set out to create a decision-making rule that could be used in ambulatory settings, such as the ED, to determine which subsets of patients suffering from GE would ultimately require antibiotics.

## Methods

Intuitively, BGE could be defined if any of the following criteria were satisfied:

1. Moderate to severe* functional disease with temperature of  ≥ 38.3 °C (≥ 101°F) and ≥ 3 days’ duration of symptoms
  *Disease severity is defined as
  Severe — total disability due to diarrhea
  Moderate — able to function, but with forced change in activities due to illness
2. Traveler’s diarrhea
3. Dysentery defined as passage of blood (in the absence of hemorrhoids) with watery stools
4. Patients presenting with watery diarrhea and having a positive bacterial stool culture [1]

We collated all patients referred to the emergency department of Ng Teng Fong General Hospital (Singapore) with symptoms consistent with gastroenteritis, who then had stool cultures performed and subdivided them into two groups:

A Those with bacterial culture–positive GE (BGE)
B Those with bacterial culture–negative GE (NBGE)

We concede that the diagnostic yield of stool cultures is low, with Slutsker et al. [5] finding only 5.6% of cultures producing bacterial isolates and other studies demonstrating yields as low as 1.5% [6]. Thus, rather than subdividing our patients into bacterial gastroenteritis and non-bacterial gastroenteritis, we used stool cultures to define *bacterial culture–positive gastroenteritis* (patients with GE with positive stool cultures as a proxy for BGE) and those with *bacterial culture–negative gastroenteritis* (patients with GE with stool cultures negative for bacteria as a proxy for NBGE).

We then performed a multivariate logistic regression analysis to derive a clinical decision-making rule that could be utilized by physicians in ambulatory care settings, such as the ED, to diagnose BGE and thus direct antibiotic prescribing practices.

A retrospective case review was performed, wherein all cases who presented acutely with infectious GE symptoms to the ED of Ng Teng Fong General Hospital (Singapore) in a two-year period between July 2015 and June 2017 and subsequently had stool cultures performed, were collated. The diagnosis of GE was next corroborated independently by both an ED physician and a gastroenterologist by analyzing case specifics, which included both (1) history of presenting illness (with the cardinal presenting symptoms being vomiting, diarrhea, abdominal pain and fever in various combinations) and (2) physical examination. Cases considered incongruent with the diagnosis of GE were excluded from our study.

Next, the data of all included cases was gathered from the review of electronic medical records (EPIC electronic case database) based on a specific questionnaire, which we constructed and then entered into an Excel spreadsheet. The clinical parameters analyzed included patient’s comorbid conditions, physical examination findings, historical markers, point-of-care and radiographic tests and other laboratory work. We then used a multi-variate logistic regression analysis on each group (bacterial culture–positive GE and bacterial culture–negative GE) to elucidate clinical criteria with the highest yield for predicting BGE. The ultimate objective was to incorporate these high yield criteria into a multi-component clinical prediction score or rule, which would be able to aid ambulatory care physicians with regard to diagnosing and treating BGE, by prescribing antibiotics judiciously.

Exclusion criteria were clearly defined as previous recent antibiotic use, urinary tract infection (UTI) symptoms, prolonged recent inpatient stay, chronic episodes of diarrhea, such as seen in irritable bowel syndrome, GI neoplasias or bleeding and diarrhea related to inflammatory bowel disease, such as Crohn’s or ulcerative colitis, and the incongruence of clinical notes with diagnosis of GE, as stated above. Pediatric patients were also excluded. Ethics approval from the National Health Group Domain Specific Review Board was obtained for the collection and analysis of data.

**Fig. 1:**
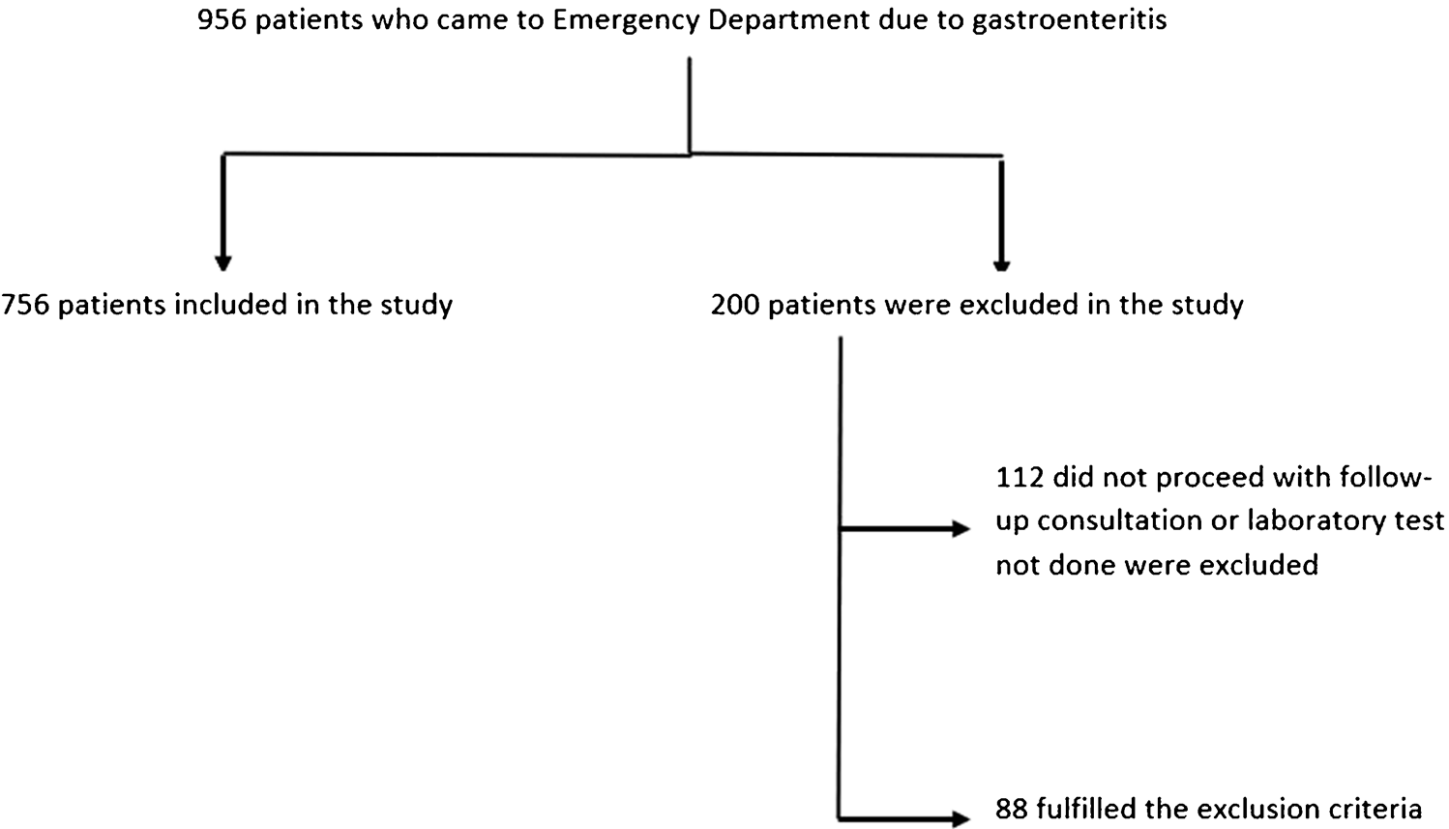
Flow chart of the study participants

A breakdown of study participants is provided in Fig. 1.

## Results

A total of 756 patients were included in our study. The mean age was 52 years, of which, 52% were female and 48% male, respectively. A total of 99.9% of our patients reported diarrhea as the main symptom, with 77.5% reporting watery type of stools with a median number of seven episodes of diarrhea per day being reported. Additionally, abdominal pain was reported by 67.2%, while 64.1% and 61% had vomiting and nausea, respectively. A majority of the subjects (*n* = 611, 80.8%) were BGE negative on stool culture.

The subjects had a median temperature of 37.2°C, with about half (49.1%) reporting fever subjectively prior to coming to the ED. Median C-reactive protein (CRP) was 51.9 mmol/L, with a range of 12.9 – 140.5 mmol/L (normal value was up to 5 mmol/L). Serum sodium median was 136 mmol/L (normal range was 133 – 138 mmol/L), and the median neutrophil count was 8.7 (normal range of 5.3–12.3 × 10^9^/L).

## Statistical analysis

Categorical variables were reported using the Chi-square test. Normally distributed continuous variables were reported as means (standard deviation, [SD]) and were compared using the Student’s *t* test and ANOVA test. Non-parametric data was reported as medians (interquartile range, [IQR]) and compared using the Mann–Whitney *U* test. To determine factors independently associated with positive BGE, variables with *p* < 0.2 on univariate analysis were entered into a multi-variable logistic regression model. The Hosmer–Lemeshow test was performed for model calibration to assess agreement between predicted and observed event rates. All tests were two-sided and statistical significance was set at *p* < 0.05. Statistical analysis was conducted using Statistical Package for Social Sciences (SPSS) (version 23.0 SPSS Inc., Chicago, IL, USA). Table 1 shows the patient’s characteristics along with key historical and examination features as well as laboratory indices.

**Table 1:** Demographics, symptoms, physical examination features and key investigations

	All patients (*n* = 756)	BGE negative (*n* = 611)	BGE positive (*n* = 145)	*p*-value
Patient demographics				
Age (years)	52.11 ± 20.06	52.48 ± 19.81	50.58 ± 21.09	0.306
Female	396 (52.4%)	323 (52.9%)	73 (50.3%)	0.572
Presentation characteristics including symptoms, signs, and contact history				
Diarrhea	749 (99.9%)	605 (100.0%)	144 (99.3%)	0.153
Blood in stool	21 (2.8%)	20 (3.3%)	1 (0.7%)	0.103
Mucous with stool	26 (3.4%)	22 (3.6%)	4 (2.8%)	0.801
Nausea	460 (61.0%)	365 (59.9%)	95 (65.5%)	0.215
Vomiting	484 (64.1%)	398 (65.2%)	86 (59.3%)	0.180
Abdominal pain	501 (67.2%)	402 (66.7%)	99 (69.7%)	0.486
Fever	368 (49.1%)	268 (44.3%)	100 (69.0%)	< 0.001*
Myalgia	42 (7.4%)	34 (7.2%)	8 (8.1%)	0.765
Travel history	112 (15.6%)	93 (16.0%)	19 (13.9%)	0.540
Sick contacts with gastroenteritis	105 (14.5%)	83 (14.2%)	22 (15.7%)	0.639
Recent gatherings with communal eating	58 (8.1%)	43 (7.4%)	15 (10.8%)	0.187
Presence for signs of dehydration	385 (54.5%)	310 (54.0%)	75 (56.8%)	0.559
Abdominal tenderness	240 (31.8%)	190 (31.1%)	50 (34.5%)	0.438
Number of episodes (per day)	7.0 (4.0–10.0)	7.0 (4.0–10.0)	8.0 (5.0–10.0)	0.209
Duration (days)	2.0 (1.0–3.0)	2.0 (1.0–3.0)	2.0 (1.3–3.0)	0.136
Number of vomiting episodes	5.0 (3.0–10.0)	5.0 (3.0–10.0)	5.0 (3.0–10.0)	0.753
Nature of stool being watery	586 (77.5%)	458 (75.0%)	128 (88.3%)	0.001*
Vital signs parameters				
Heart rate (BPM)	90.03 ± 19.52	90.03 ± 19.84	90.03 ± 18.21	0.999
Systolic blood pressure (mmHg)	124.08 ± 22.63	124.27 ± 22.67	123.28 ± 22.55	0.635
Diastolic blood pressure (mmHg)	72.48 ± 13.70	72.33 ± 13.49	73.11 ± 14.59	0.537
Mean arterial pressure (mmHg)	89.68 ± 14.62	89.64 ± 14.49	89.83 ± 15.22	0.889
Digital rectal examination blood in stool	446 (59.0%)	358 (58.6%)	88 (60.7%)	0.644
Investigations—laboratory indices and radiography				
Hematocrit (%)	40.78 ± 6.88	40.50 ± 6.84	41.96 ± 6.97	0.230
Hemoglobin (g/L)	13.66 ± 3.01	13.57 ± 2.96	14.04 ± 3.19	0.192
Temperature (°C)	37.2 (36.6–38.1)	37.1 (36.6–38.0)	37.3 (36.7–38.4)	0.013*
White blood cells count (× 10^9^/L)	10.9 (7.4–14.4)	11.2 (7.8–14.6)	9.3 (6.4–14.0)	0.093
Neutrophil count (× 10^9^/L)	8.7 (5.3–12.3)	9.1 (5.5–12.4)	7.4 (4.7–12.1)	0.017*
C-reactive protein (mg/L)	51.9 (12.9–140.5)	44.4 (10.7–132.4)	95.1 (27.3–149.4)	0.008*
Serum urea (mmol/L)	5.6 (3.9–8.6)	5.5 (3.9–8.5)	5.8 (3.8–10.1)	0.760
Serum creatinine (μmol/L)	84.0 (63.0–127.0)	82.0 (63.0–122.5)	94 (65.0–149.0)	0.085
Serum sodium (mmol/L)	136.0 (133.0–138.0)	136.0 (134.0–139.0)	135.0 (131.0–138.0)	0.001*
Serum potassium (mEq/L)	3.9 (3.5–4.2)	3.9 (3.5–4.2)	3.8 (3.5–4.2)	0.157
Serum bicarbonate (mEq/L)	20.0 (18.0–22.0)	20.0 (18.0–22.0)	19.0 (17.5–22.0)	0.278
Serum lactate (mmol/L)	1.9 (1.4–2.9)	1.9 (1.4–2.9)	1.7 (1.2–2.7)	0.257
Anion gap (mEq/L)	15.7 (14.1–18.0)	15.7 (14.0–18.0)	15.9 (14.5–18.2)	0.294
Serum chloride (mmol/L)	104.0 (101.0–107.0)	104.0 (101.5–107.0)	103.0 (99.0–106.5)	0.005*
Positive blood culture results	475 (62.8%)	387 (63.3%)	88 (60.7%)	0.553
Positive stool culture results	147 (19.4%)	2 (0.3%)	145 (100.0%)	< 0.001*
Positive findings in CXR or CT report	652 (86.2%)	521 (85.3%)	131 (90.3%)	0.111

Values are expressed in number (percentage); mean ± standard deviation; and median (interquartile range)

*BGE* bacterial gastroenteritis, *BPM* beats per minute; *CXR* chest X-ray; *CT* computed tomography

^*^p-value < 0.05

A non-parametric test was performed for the laboratory results, which was followed by proportional Chi-square test for the categorical variables with a *p*-value < 0.05, deemed significant.

The following variables were deemed significant (*p* < 0.1) in correctly classifying patients having bacterial gastroenteritis:

- Serum sodium
- Serum chloride
- C-reactive protein
- White blood cell count
- Neutrophil count
- Objective fever
- Watery stool

The Youden Index was used to maximize the sum of sensitivity and specificity to determine the cut-off for each continuous variable. We further identified six variables, which were of particular interest to this study and a rank score model was performed with AUROC for the scoring system (range 0 – 8) is 0.812 + 0.016 (95% CI: 0.780–0.843), *p*-value < 0.001. White blood cells were excluded, as these might not be of clinical interest in differentiating bacterial and viral load in GE.

In clinical practice, a C-reactive protein of over 10 mg/L is considered elevated. Based on the study, 79.8% of BGE patients had a CRP of over 10 mg/l, which carries the most weightage of all identified variables. A second cut-off of 16.6 mg/L yielded a sensitivity and specificity of 70.3% and 76.5%, respectively, while a cut-off  >25 mg/L yielded the highest specificity of 98.2% and the greatest positive likelihood ratio for identifying patients with BGE.

The six variables with their cut-off values are as follows:

- Serum sodium (< 135 mmol/L)—1 point
- C-reactive protein
- 10 to 16.5 mg/L—1 point
- 16.6 mg/Lme 24.9 mg/L—2 points
-  > 25 mg/L—3 points
- Neutrophil count (> 8 × 10^9^/l)—1 point
- Objective fever (yes)—1 point
- Subjective feeling of being febrile (yes)—1 point
- Watery stool (yes)—1 point

These six variables were integrated into a composite score, which we termed the BGE score.

As illustrated in Table 2, only four patients of 448 (0.9%), who scored 0–2 points in the BGE score, had BGE, while 20 cases of the 167 patients (12%) with a score of 3 or 4 were found to have BGE. Finally, 121 patients of 141 (85.8%), who scored 5 or more points, were found to have BGE.

**Table 2:** Number of patients diagnosed with bacterial gastroenteritis (BGE) and without bacterial gastroenteritis according to the BGE scoring system

BGE score	Number of BGE	Number of not BGE
0	0 (0.0%)	101 (0.0%)
1	0 (0.0%)	106 (0.0%)
2	4 (1.7%)	237 (98.3%)
3	3 (3.1%)	94 (96.9%)
4	17 (24.3%)	53 (75.7%)
5	24 (72.7%)	9 (27.3%)
6	49 (82.7%)	11 (18.3%)
7	39 (100.0%)	0 (0.0%)
8	9 (100.0%)	0 (0.0%)

*BGE* bacterial gastroenteritis

Thus, based on these results, the BGE score was stratified into low (0 – 2 points), intermediate (3 – 4 points) and high-risk (≥ 5 points) groups, based on the likelihood of diagnosing BGE.

Table 3 summarizes the various accuracy measures for the different cut-offs in the high-risk category of the BGE score. A BGE score of  ≥ 5 points yielded a positive predictive value (PPV) of 86.5%, a score of  ≥ 6 presented a PPV of 91.1%, and a score of  ≥ 7 yielded a PPV of 100%.

**Table 3:** Sensitivity, specificity, negative predictive value and positive predictive value for different cutoffs of the bacterial gastroenteritis (BGE) scoring system showing high specificity and positive predictive value for bacterial gastroenteritis with a BGE score of  ≥ 5

BGE score	Sensitivity (%)	Specificity (%)	NPV (%)	PPV (%)	LR +	LR −	Youden Index
5	70.8	91.7	84.4	86.5	8.3	0.32	0.625
6	62.1	95.8	75.4	91.1	14.8	0.40	0.579
7	43.5	100.0	65.0	100.0	∞	0.57	0.435
8	10.6	100.0	55.5	100.0	∞	0.89	0.106

*BGE* bacterial gastroenteritis, *PPV* positive predictive value, *NPV* negative predictive value, *LR* + positive likelihood ratio, *LR* negative likelihood ratio

Similarly, no cases of BGE would be missed with a cut-off ≤ 2 (NPV of 100%). These findings could be useful to clinicians in diagnosing BGE based on commonly performed simple laboratory tests and routine elements of a patient’s clinical presentation.

## Discussion

The pathogenesis of GE is multi-factorial, but often occurs when microbial virulence overwhelms normal host defenses, with the normally acidic pH of the stomach and colon serving as an effective antimicrobial defense mechanism. However, certain situations impair this aforementioned defense. These include achlorhydric states (i.e. those caused by antacids, histamine-2 [H2] blockers, gastric surgery) and decreased colonic anaerobic flora, which impair intestinal immune homeostasis. Similarly, an alteration of normal bowel flora can create a biologic void that is filled by potential pathogens. This occurs most commonly after antibiotic administration. Additionally, hypomotility states may result in colonization by pathogens, especially in the proximal small bowel, where motility is the major mechanism responsible for the removal of organisms. Hypomotility states are associated with anti-peristaltic agents (e.g. opiates, diphenoxylate and atropine [Lomotil], loperamide) or anomalous anatomy (e.g. fistulae, diverticula, anti-peristaltic afferent loops) and is inherent in disorders such as diabetes mellitus or scleroderma. Importantly, an immunocompromized host is more susceptible to infection, as evidenced by the wide spectrum of diarrheal pathogens in patients with acquired immunodeficiency syndrome (AIDS) [2]. Finally, a large inoculation of a pathogenic microbe (which may include viruses, bacteria, or protozoa) may overwhelm a host’s capacity to mount an effective defense.

Diarrhea is one of the most common presenting symptoms, for which patients seek medical care. In the developed world, it is one of the most common reasons for missing work (morbidity), while in the developing world, it is a leading cause for mortality. An estimated 179 million cases of acute GE occur every year in the US. Of these patients, a vast majority (80% to 85%) do not seek medical attention and only a minority (1% to 2%) require hospital admission [7]. Diarrheal diseases can quickly reach epidemic proportions, rapidly overwhelming public health systems in even the most-advanced societies.

Very often, gastroenteritis is underreported in the adult population. Each year, gastroenteritis in adults accounts for 8 million doctor visits and 2,50,000 hospitalizations. Episodes of gastroenteritis do not often occur at random, but do usually take place in outbreaks. Traveler’s diarrhea affects 20% to 50% of people traveling from industrialized to developing countries [8–11].

Gastroenteritis is a major cause for death worldwide, but is of particular concern in developing countries, where sanitation and access to medical care are limited. However, by no means is this exclusively a problem of the developing world. The Centers for Disease Control and Prevention (CDC) (US) reported that enteritis deaths doubled in the US between 1999 and 2007, from about 7000 to 17,000. In 2015, there were two billion cases of gastroenteritis, resulting in a staggering 1.3 million deaths globally [12, 13]. Children and those in the developing world are affected the most [14].

The management of GE requires a comprehensive clinical evaluation, an accurate diagnosis and then appropriate treatment, both supportive and antimicrobial. Patient history and examination alone have been found to be notoriously poor at differentiating BGE from NBGE. However, epidemiological studies have noted that the prevalence of BGE is approximately one-third that of NBGE.

Management of GE is principally supportive, while the goals of pharmacotherapy are essentially to reduce morbidity and prevent complications. It is well-recognized that many cases are self-limited and require no antimicrobial therapy. However, many antibiotic recommendations have been established to target specific bacterial serologies, e.g, *Campylobacter* species—erythromycin is said to shorten the illness duration and shedding with maximal effect, when therapy is used within four days from the onset of symptoms [15].

However, we feel that this is not useful for the frontline ED physician or equivalent as the causative agent is almost never known. Hence, it is important to come up with a practical clinical decision rule (CDR) that may guide appropriate antibiotic use. Established guidelines addressing the prescribing of antibiotics in GE have merely suggested its use often advising testing, which is often impractical [﻿16]. Most agree that the intuitive, but rather rare, findings of a septic patient or one with blood and mucous in the stool necessitate antibiotic administration in GE, especially in immunocompromized hosts. This has resulted in variant practices principally due to a lack of clarity among practicing clinicians using such guidelines.

Additionally, a cohort study conducted in the primary healthcare sector in the UK to determine antibiotic prescribing practices among GPs, between 2013 and 2014, found that empirical treatment was more frequent (enacted in 55% of all prescribed cases) compared with targeted treatment and that too was associated mostly with non-Clinical Practice Guideline (CPG)–recommended antibiotics. It highlighted the need for more judicious prescribing practices via a targeted approach [17].

A CDR would certainly provide an avenue to allow frontline physicians to provide such targeted antibiotic prescribing in adult gastroenteritis patients presenting to ambulatory care settings.

With the data above in Table 4, we suggest using the BGE scoring system to delineate the need for empirical antibiotics in adult patients presenting with gastroenteritis, specifically at a cut-off  ≥ 5 points.

**Table 4:** Recommended scoring system for bacterial gastroenteritis

*Variables*	*Range*	*Points*
Serum sodium	< 135 mmol/l	*1*
C-reactive protein	10–16.5 mg/l	*1*
	16.6–24.9 mg/l	*2*
	> 25 mg/l	*3*
Neutrophil	> 8.0 × 10^9^/l	*1*
Temperature	> 37.5 °C	*1*
Febrile	Yes	*1*
Watery stool	Yes	*1*
Total	≥ 5 points determine the high possibility of positive BGE	8

*BGE* bacterial gastroenteritis

Clinical decision rules (CDRs), also known as Decision “Instruments,” are evidence-based tools designed to help clinicians make bedside diagnostic and therapeutic decisions for common complaints and presentations. It is important to state that CDRs are in their purest form “decision aids” and are not meant to replace critical thinking or physician gestalt especially from experienced practitioners.

A useful CDR must possess three main criteria. Firstly, the condition needs to be relatively common. Secondly, there must also be a perceived inefficiency or clinical variability in practice with regard to the workup of the patient. For example, the inefficiency can be an over-use or under-use of a particular resource (imaging, blood tests, antibiotic use, etc.), which, given a lack of evidence, physicians have different approaches to. Finally, the clinical question that leads to the inefficiency needs to be answerable with only a handful of clinical variables [18].

We believe that our study caters to all three criteria. Our aim is to derive a multi-component CDR, predicated on simplistic criteria, which are readily available in an ED or similar ambulatory setting, which will guide physicians’ antibiotic prescribing practices in adult patients presenting with the extremely common condition of gastroenteritis.

An ideal CDR must be of practical utility and relatively simplistic, if it is to be benefit to the practitioner who uses it. The use of simple criteria will result in greater uptake and usability, especially in a busy clinical environment such as the ED. Conversely, if a CDR is developed that has too many complicated variables, it is unlikely to be applied in a busy ED or similar setting.

The development of a CDR generally involves three distinct phases: derivation, validation, and implementation. This study’s purpose aims to derive a CDR for defining bacterial gastroenteritis (phase 1), with validation and implementation our future goals. Numerous implementation studies have demonstrated that the implementation of CDRs in the ED reduced the use of resources [19].

**Fig. 2:**
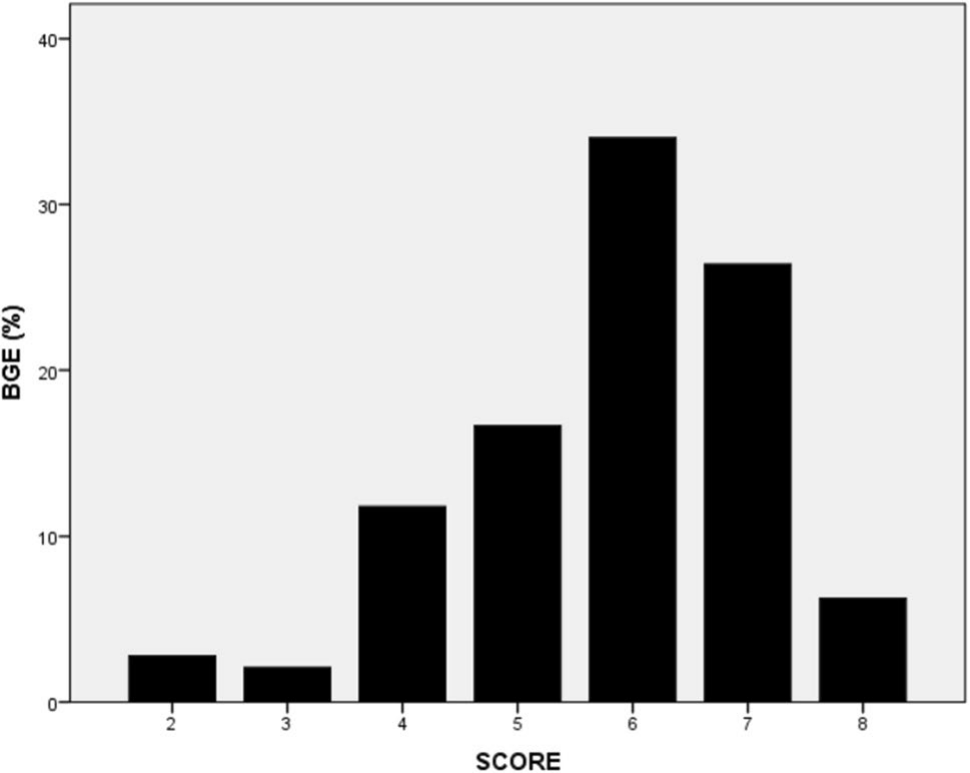
Rate of bacterial gastroenteritis (BGE %) vs. BGE score in 756 participants

Based on the rate of BGE associated with each score as shown in Fig. 2, a score 0 – 2 points suggests a low risk (0.9%) of BGE. A score of 3 – 4 points confers an intermediate risk of 12.0%, and a score of 5 – 8 points confers a high risk of 85.7%. A cut-off of  ≥ 5 points may be used to predict culture-positive BGE with a 75% sensitivity and 75% specificity. The AUROC for the scoring system (range 0 – 8) was 0.812 ± 0.016 (95% CI: 0.780–0.843), *p*-value < 0.001. The negative predictive value for the rank score model of  ≥ 5 was 75.4%, while its positive predictive value was 75.4%. The specificity for the rank score model of  ≥ 5 was 69.5%, and its sensitivity 86.1%.

Thus, we suggest using a BGE score of  ≥ 5 points (high risk for BGE), after shared decision-making (SDM), to prescribe antibiotics to adult patients presenting with gastroenteritis.

The most common antibiotic prescribed for BGE in adults is a three-day course of ciprofloxacin, which costs approximately Singapore dollar (SGD) $6 per course (or United States Dollar [USD] $4.50). The financial magnitude of the problem is thus potentially astounding. Assuming a conservative 30% of BGE presenting to ambulatory care settings received antibiotics worldwide based on physician judgment but the use of a decision-making rule (such as the BGE score, which allowed for a more accurate diagnosis of BGE and thus more judicious antibiotic prescribing) decreased the antibiotic prescriptions to 18.5% (as per our study), this would result in a sizable total savings of SGD $5.52 million worldwide (using the above-mentioned incidence of 8 million doctor visits per year). Economics aside, another important benefit would be to save these select patients (NBGE) from the adverse effects that are the inevitable concomitance of needless antibiotic administration, including potential adverse drug reactions and antibiotic resistance.

While this is a pilot study, which will require further validation with a larger sample size, our proposed decision-making rule will potentially serve to improve the diagnosis of BGE, especially in adult patients presenting with GE to ambulatory care settings. This in turn would reduce unnecessary prescribing of antibiotics, which would thus reduce antibiotic-associated adverse events and resistance as well as save costs worldwide.

## Data Availability

The datasets used and/or analyzed during the current study are available from the corresponding author on reasonable request.

## Declarations

### Disclaimer

The authors are solely responsible for the data and the contents of the paper. In no way is the Honorary Editor-in-Chief, Editorial Board Members, the Indian Society of Gastroenterology or the printer/publishers responsible for the results/findings and content of this article.
